# Quorum Sensing Influences *Vibrio harveyi* Growth Rates in a Manner Not Fully Accounted For by the Marker Effect of Bioluminescence

**DOI:** 10.1371/journal.pone.0001671

**Published:** 2008-02-27

**Authors:** Zeena E. Nackerdien, Alexander Keynan, Bonnie L. Bassler, Joshua Lederberg, David S. Thaler

**Affiliations:** 1 Raymond and Beverly Sackler Laboratory of Molecular Genetics and Informatics, Rockefeller University, New York, New York, United States of America; 2 Department of Biological Chemistry, Institute of Life Sciences, The Hebrew University of Jerusalem, Jerusalem, Israel; 3 Department of Molecular Biology, Howard Hughes Medical Institute, Princeton University, Princeton, New Jersey, United States of America; Newcastle University, United Kingdom

## Abstract

**Background:**

The light-emitting Vibrios provide excellent material for studying the interaction of cellular communication with growth rate because bioluminescence is a convenient marker for quorum sensing. However, the use of bioluminescence as a marker is complicated because bioluminescence itself may affect growth rate, e.g. by diverting energy.

**Methodology/Principal Findings:**

The marker effect was explored via growth rate studies in isogenic *Vibrio harveyi* (*Vh*) strains altered in quorum sensing on the one hand, and bioluminescence on the other. By hypothesis, growth rate is energy limited: mutants deficient in quorum sensing grow faster because wild type quorum sensing unleashes bioluminescence and bioluminescence diverts energy. Findings reported here confirm a role for bioluminescence in limiting *Vh* growth rate, at least under the conditions tested. However, the results argue that the bioluminescence is insufficient to explain the relationship of growth rate and quorum sensing in *Vh*. A *Vh* mutant null for all genes encoding the bioluminescence pathway grew faster than wild type but not as fast as null mutants in quorum sensing. *Vh* quorum sensing mutants showed altered growth rates that do not always rank with their relative increase or decrease in bioluminescence. In addition, the cell-free culture fluids of a rapidly growing *Vibrio parahaemolyticus* (*Vp*) strain increased the growth rate of wild type *Vh* without significantly altering *Vh*'s bioluminescence. The same cell-free culture fluid increased the bioluminescence of *Vh* quorum mutants.

**Conclusions/Significance:**

The effect of quorum sensing on *Vh* growth rate can be either positive or negative and includes both bioluminescence-dependent and independent components. Bioluminescence tends to slow growth rate but not enough to account for the effects of quorum sensing on growth rate.

## Introduction

A great number of nutritional and environmental factors are known to influence the growth rate of bacteria. Lately some information has been published consistent with the idea that the growth rate of bacteria may not be only determined by factors of nutrition and the environment, but that it might be “self controlled” in bacterial populations by cellular communication or quorum sensing [1]–[3].

Growth and communication are fundamental processes in biology and the study of their interaction is of intrinsic interest. It is now understood that most bacteria contain genes coding for the formation of density-dependent quorum sensing systems [4]–[7]. Quorum sensing is well characterized in the genus Vibrio [7]–[13]. These cited studies were mostly aimed at understanding the effect of quorum sensing on light emission and did not specifically address themselves to the influence of quorum sensing on growth rate.

The near miraculous harmonization of metabolism required for balanced growth is a classic problem of biology [14] and new methodology promises to reinvigorate its exploration [15], [16]. By hypothesis, the achievement of balanced growth is especially demanding at higher growth rates. The genus Vibrio harbors some of the fastest growing bacteria known [17], [18], and thereby provides potential material for study of the most extreme demands and phenotypes of rapid bacterial cellular growth.

Although the biochemical mechanism and quorum-dependent regulation of bacterial bioluminescence are well studied [9], [12], [13], there are discordant reports regarding the influence of light production itself on the growth rates of luminescent Vibrios. The slower growth rates of bright Vibrios relative to dark mutants in some studies are in keeping with the energy sink hypothesis which states that the energetic costs of light production slows growth rate [10]–[13]. However, other studies found that the energetic drain of luminescence had no influence when comparing growth rates of bright Vibrios and dark mutants ([19] and references therein). In addition, there are reports that mutations in single quorum genes cause no change in growth rate but might reduce final growth yield of a *Vibrio fischeri* quorum mutant to 75% of wild type levels [20]. Hence the inter-relationship of luminescence and growth rate has not yet been solved. This paper provides evidence that quorum sensing also influences growth rate in a manner that cannot fully be accounted for by the energy drain of light emission alone.

**Figure 1:**
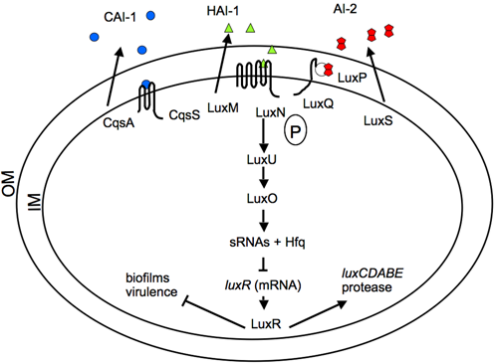
The overall structure of the *Vh* quorum sensing circuit showing quorum genes mutated in this work. *Vh* has three quorum sensing systems that regulate the genes specifying biofilm formation, a type III secretion system, a secreted metalloprotease, bioluminescence (*luxCDABE*) and other quorum sensing activated- and repressed-genes. System 1 is composed of an autoinducer, HAI-1 (triangles) and its cognate sensor, LuxN. System 2 is composed of autoinducer-2 (pentagons) and the sensor, LuxPQ. System 3 is composed of an intragenera signal, CAI-1 and its cognate sensor, CqsS. At low cell densities, autoinducer signals and phosphate (P) flow act in a negative manner to repress autoinducer genes, and at high cell densities the phosphate flow is reversed and signals act in a positive manner to activate some of the quorum-dependent genes e.g. luciferase. See Introduction for further details. OM, outer membrane; IM, inner membrane.

Growth rate and bioluminescence experiments were carried out on an isogenic set of *Vibrio harveyi* mutants. The three autoinducer-sensor systems comprising the *Vh* system are described in Figure 1: Autoinducer-1 (denoted HAI-1 for *V. harveyi* autoinducer-1; N- (3′-hydroxybutanoyl) homoserine lactone), Autoinducer-2 (AI-2, a furanosyl borate diester) and the third autoinducer called CAI-1 (for Cholerae Autoinducer-1;(S)-3-hydroxytridecan-4-one [21]) have been studied in detail in *Vibrio harveyi* (see Figure 1), where they are involved in the regulation of bioluminescence and dozens of other traits. HAI-1 is produced by LuxM and is detected by LuxN. LuxM and HAI-1 activity appears to be restricted to *Vh* and the closely related species *Vibrio parahaemolyticus* (*Vp*), indicating that this signal is relatively species-specific. Two proteins, LuxP and LuxQ, function together to detect AI-2 (unborated precursor synthesized by LuxS). LuxS and AI-2 production are widespread in the bacterial world, and AI-2 is proposed to be an inter-species communication signal. CAI-1 is made by CqsA and interacts with its cognate sensor, CqsS. The CAI-1-CqsS system is found predominantly in Vibrios, suggesting it could be a genus-specific system. CAI-1 differs from HAI-1 and AI-2 by being detected at extremely low cell densities [22]. The cell-density-dependent information supplied by all three signals is channeled into the cytoplasm by a phosphorylation cascade that converges on a protein called LuxO [23]. LuxO negatively regulates the expression of *luxR*, encoding the master transcription factor, by activating the expression of multiple small RNAs (sRNAs) that together with the chaperone Hfq, destabilize the *luxR* mRNA. At high cell densities, LuxO is inactive and so it cannot promote expression of the genes encoding the sRNAs, the *luxR* mRNA is stabilized, LuxR is produced, and it initiates quorum sensing-controlled behaviors such as expression of the luciferase operon (*luxCDABEGH*). The luciferase enzyme is composed of two proteins, LuxA and LuxB, while additional proteins, LuxC, LuxD and LuxE are responsible for recycling the aldehyde substrate (for a comprehensive review, see [13]).

This study investigated the luminescence-growth relationships for wild type *Vibrio harveyi* and an isogenic set of strains mutated at steps throughout the quorum sensing pathway. The results do not categorically disprove the energy sink hypothesis, but indicate that the modulation of bioluminescence is insufficient to account for the observed influence of quorum sensing on the growth rate of *Vibrio harveyi*.

*Vibrio parahaemolyticus* (*Vp*) produces quorum factors that can stimulate luminescence in *Vibrio harveyi* quorum sensing mutants [9], a result confirmed in this study. In addition, this study found that cell-free culture fluids from a fast-growing *Vibrio parahaemolyticus* strain increased the growth rate of wild type *Vibrio harveyi*.

## Results

### Bioluminescence, quorum sensing and growth rate in Vibrios

*Vh* luminescence and growth rate were measured in mini-batch cultures using a microtiter plate format. Growth rates were calculated from the slopes of OD as described in Materials and Methods and converted to doublings per hour. For example, a culture that doubles once per 60 minutes has a growth rate of 1.0/H; a culture with a doubling time of 30 minutes has a specific growth rate of 2.0/H.

**Figure 2:**
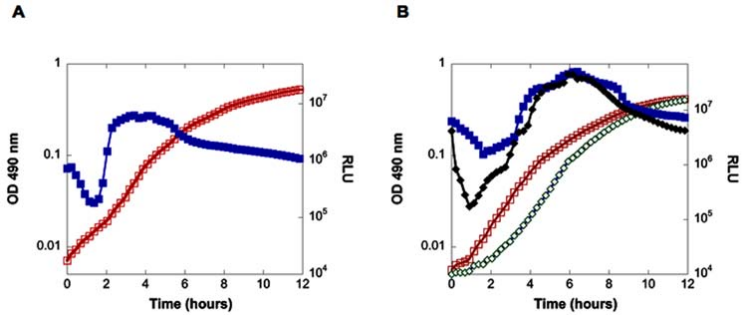
Bioluminescence-growth relationships for the *Vh* strains that served as wild type references for comparison with mutants in quorum sensing and bioluminescence. The isogenic, wild type *Vh* strains, (A) BB120, growth rate (□) and RLU or relative light units expressed as counts per second/OD (*v*) and (B) BB866 (WT::Tn5) in the absence (growth rate (□) and relative light units, (*v*)) and presence of the antibiotic, kanamycin (growth rate, ⋄ and Relative Light Units, ⧫).

The growth curves of two isogenic wild type *Vh* strains are shown in Figure 2. Both strains are wild type with respect to the three known quorum sensing autoinducer receptors (LuxPQ, LuxN, and CqsS) and they respond to all three autoinducers (HAI-1, AI-2, and CAI-1). Strain BB120 is the wild type base strain for these studies. Strain BB866 is *V. harveyi* BB120, harboring a Tn5 insertion upstream of *luxPQ*. Strain BB866 was constructed as described in other studies and the inserted transposon has no polar effects on neighboring genes [24]. Strains BB120 and BB866 grew at growth rates of 1.0/H in similarly inoculated wells.

In *E. coli*, the bleomycin resistance gene of Tn5 confers a growth advantage even in the absence of antibiotics [25]. To learn if Tn5 or kanamycin might play a role in the growth rate of *Vh* in this study, strain BB866 was further examined in the absence and presence of kanamycin. An influence of Tn5 and kanamycin was evident on the lag phase, but not on the exponential phase growth rates of strain BB866 (Figure 2B). Strains BB120 (wild type with no Tn5) and BB866 displayed the same bioluminescence phenotypes, i.e. dilution of the cultures caused the autoinducer levels to decline and bioluminescence per cell to be drastically reduced. However, during further growth, released autoinducers accumulated to threshold levels, initiating light production that increased to peak-levels during late-exponential phase.

Bioluminescence is influenced by non-quorum signals, including nutrition and osmolarity [26], [27]. The influence of media dilution on the luminescence and growth phenotypes of both wild type strains was tested with two- and four-fold dilutions of Difco Marine Broth (MB) with artificial seawater (ASW). Dilution decreased the growth rates of both wild-type strains (growth rates - MB:ASW(1:2) = 0.8/H and MB:ASW (1:4) = 0.7/H), without affecting the peak relative light units ( approximately 10^7^; Figure S1).

Growth rate analysis of a panel of isogenic *Vh* mutants was carried out (transposon insertion and deletion mutants as well as mutants generated partly by homologous recombination, see table 1). The wild type strain, *Vh* BB866, was used as a reference strain for comparison with isogenic *Vh* quorum mutants.

**Table 1:** Strains, Genotypes and Vectors

Strain name	Relevant Features	Reference or Source
*Vibrio harveyi*	Wild Type	[9]
BB120		
BB866	Wild Type::Tn5	[24]
BB152	*luxM*::Tn5	[9]
MM30	*luxS*::Tn5	[6]
JMH603	*cqsA*::Cm^r^	[22]
JMH634	Δ*luxM* Δ*luxS cqsA*::Cm^r^	[22]
KM664	*luxR*::Tn5	B.Bassler unpublished
BB170	*luxN*::Tn5	[9]
BB886	*luxPQ*::Tn5	[9]
JMH598	*cqsS*::Cm^r^	[22]
JMH628	Δ*luxN luxQ*::Tn5 *cqsS*::Cm^r^	[22]
BB721	*luxO*:Tn5	[23]
BB151120	*luxCDA*::Tn5*BE*	B.Bassler unpublished
*Vibrio parahaemolyticus*	Wild Type	R. Colwell, Univ. Maryland
UM4552		
Vectors		
pLAFR2	Broad-host range, *mob*, Tet^r^	[22]
pRK2013	Broad host range, *tra*	[57]

Bacterial bioluminescence is catalyzed by luciferase, an enzyme composed of heterodimeric subunits called α and ß (encoded by *luxA* and *luxB*). Eliminating luciferase genes alone prevents enzyme synthesis, but complicates the interpretation of growth rate changes because of the potential accumulation of an inhibitory aldehyde substrate [13]. The influence of bioluminescence on growth rate in a background wild type for quorum sensing was assayed in a *Vh luxCDABE* mutant (strain BB151120). This mutant grew 30% faster than the wild type strain (Growth rates are given in Table 2).

**Table 2:** Growth rates of *V. harveyi* strains in MB.

Strain name	Relevant Characteristics	Growth Rate (/H)
BB120	Wild Type	1.0±0.03
BB866	Wild Type	1.0±0.05
BB152	HAI-1^−^	1.6±0.07
MM30	AI-2^−^	1.4±0.08
JMH603	CAI-1^−^	0.9±0.08
JMH634	HAI-1^−^, AI-2^−^, CAI-1^−^	1.3±0.03
KM664	LuxR^−^	1.2±0.04
BB151120	LuxCDABE^−^	1.3±0.05
BB170	LuxN^−^	1.5±0.06
BB886	LuxPQ^−^	1.4±0.09
JMH598	CqsS^−^	0.9±0.09
JMH628	LuxN^−^,LuxPQ^−^,CqsS^−^	0.5±0.04
BB721	LuxO^−^	0.5±0.15

**Figure 3:**
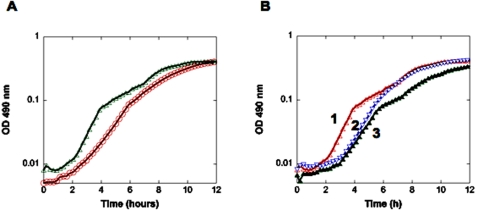
(A) Bioluminescence-growth relationships of wild type *Vh* (BB866, WT::Tn5, ○) and a BB151120 (*luxCDABE*::Tn5) mutant inactivated for all bioluminescence-associated genes. *Vh* strains were grown in the presence of antibiotics (see Materials and Methods). (B) The BB151120 mutant was further analyzed under different conditions.(▵) line 1, chromosomal mutation; (▿) line 2, strain carrying pLAFR2 vector control and (▴) line 3, strain carrying pLAFR2 with the cloned *luxCDABE* genes.

BB151120 was sham “complemented” with either an empty vector (pLAFR2) carrying the tetracycline marker or truly complemented with pLAFR2 containing a wild type *luxCDABE* allele (under control of its endogenous promoter; Figure 3B). The presence of the empty vector did not affect the luminescence phenotype and only slightly decreased the growth rate to 1.2/H. Complementation with wild type *luxCDABE* on pLAFR2 restored bioluminescence and slowed the average growth rate to 1.0/H (see Table 3). Both vectors, i.e. pLAFR2 with or without *luxCDABE,* led to a lag phase that was about 1 hour longer. It is possible that this increase lag phase is dependent on the antibiotic whose resistance is encoded on pLAFR2 and/or the replicon coordination between plasmid and chromosome play a role in increasing the lag phase.

**Table 3:** Growth rates of specific *V. harveyi* mutants complemented with wild-type alleles.

*Vh* Strain name	Mutation	Autoinducers Detected	Base Strain Growth rate (/H)	Empty Vector, pLAFR2 Growth rate (/H)	Vector, pLAFR2 with WT allele Growth rate (/H)
BB151120	*luxCDABE*	HAI-1, AI-2, CAI-1	1.3±0.05	1.2±0.02	1.0±0.02
MM30	*luxS*	HAI-1, CAI-1	1.4±0.08	1.3±0.03	1.1±0.02
BB152	*luxM*	AI-2, CAI-1	1.6±0.07	1.5±0.10	1.4±0.11
BB170	*luxN*	AI-2, CAI-1	1.5±0.06	1.3±0.03	0.9±0.03
BB886	*luxPQ*	HAI-1, CA-1	1.4±0.09	1.3±0.04	1.0±0.04

**Figure 4:**
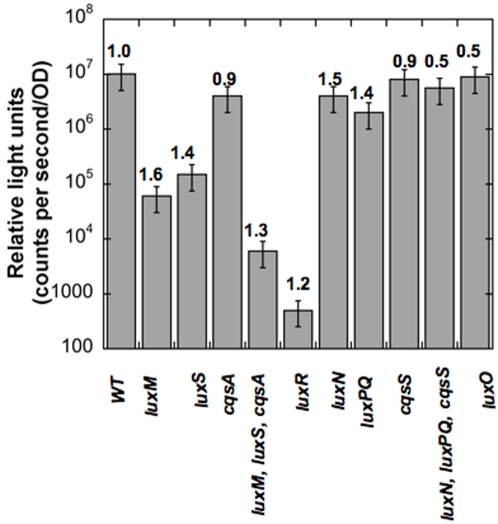
The peak bioluminescence of autoinducer synthase and sensor mutants. Bars represent Relative Light Units (RLU = counts per second/OD) and growth rates (doublings/H; from table 2) are given above each bar. The wild type strains, BB120 and BB866 (WT::Tn5) gave similar RLU values. Wild type RLU values refer to strain BB866 in this figure. Mutant strains: BB152 (*luxM*), MM30 (*luxS*), JMH603 (*cqsA*), JMH634 (*luxM*, *luxS*, *cqsA*), KM664 (*luxR*), BB170 (*luxN*), BB886 (*luxPQ*), JMH598 (*cqsS*), JMH628 (*luxN*, *luxPQ*, *cqsS*) and BB721 (*luxO*).

The quorum sensing mutants under study exhibited expected bioluminescence phenotypes, in keeping with previous findings e.g. the HAI-1-mutant produces 0.3%, the AI-2-mutant produces 3% and the CAI-1-mutant produces 33% of the light produced by the wild type, respectively (see Figure 4). If quorum sensing influenced growth rates primarily through luminescence, bright strains would always grow slowly. Indeed, two mutants exhibited growth defects in keeping with what is known about their luminescence, namely *luxO* and *luxN*, *luxPQ*, *cqsS* triple mutants (Table 2). These mutants are brighter throughout the exponential phase of growth in contrast to WT , which only reaches the same level of luminescence as these mutants at a high cell density. In addition, these mutants also mimic high cell density states because in both cases, there is no phosphorus flow through the circuit (see Figure 1) [22], [23]. An extension of the energy sink hypothesis is that dim strains might always grow faster in the following order (Figure 4): *luxR*>*luxMluxScqsA*>*luxM*>*luxS*>*cqsA*. The atypical growth rates observed for dim strains (Table 2 and Figure 4) can only be partly accounted for by considering signaling strengths. The third autoinducer production/detection system, composed of the autoinducer-sensor pair, CAI-1-CqsS, had previously been shown to have the weakest signaling strength [22], and consistent with this, the growth rates of strains carrying single mutations in this system were essentially unchanged compared to the wild type (see table 2). However, the fastest growth rates were observed for two mutants with opposite luminescence phenotypes and signaling strengths, namely the *luxM* (HAI-1^−^, mimics low cell density due to permanent phosphorylation of LuxO by LuxN [9], [22], [23]) and *luxN* (sensor1-; mimics high cell density due to the lack of phosphorylation of LuxO [9], [22], [23]) mutants (Table 2). A copy of *luxM* complemented the luminescence (data not shown) but not the growth phenotype (Table 3). Successful complementation of the remaining quorum mutants (Table 3) are consistent with the idea that growth rate changes were consequent to quorum sensing.

### *Vp* secretes extracellular factors that influences *Vh* growth rates

**Figure 5:**
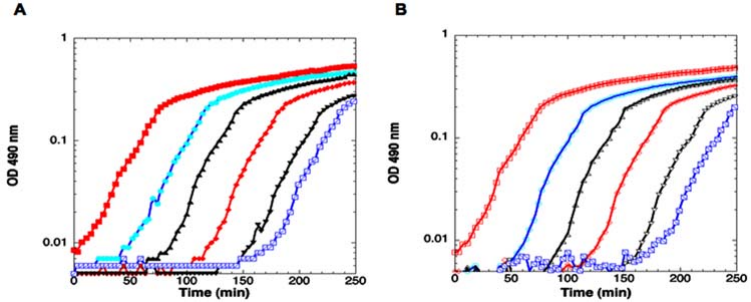
The influence of inoculation dilution and cell-free culture fluids from *Vp* cultures on *Vp* growth rates. Cultures were grown in pre-warmed MB at 37 °C. Representative graphs: (A) UM4552 culture was diluted and growth curves are starting at different cell densities. The dilutions are: (▪) 1∶10, (•) 1∶10^2^, (▴) 1∶10^3^, (♦) 1∶10^4^, (▾) 1∶10^5^ and (Open Square with Diagonal Line Running from Upper right corner to Lower left corner) 1∶10^6^. (B) UM4552 culture was diluted and growth curves are starting at different cell densities. The dilutions are the same as in panel A. The table shows the corresponding growth rates for each of the dilutions.

A candidate for a rapidly growing isolate of the marine bacterium *Vp* was the kind gift of Rita Colwell (UM4552; see strain table 1). The rapid growth rate was confirmed with manual readings from flask cultures maintained at 37°C and conventional spectrophotometry and flow cytometry (data not shown) as well as the microtiter plate method for which readings were taken every 2.5 minutes. UM4552, like other reported *Vp* isolates, contained all the *Vh*-like quorum genes as judged by PCR (Table S1). In addition, its growth rate increased about 1.7-fold when an exponential UM4552 culture was diluted up to 1∶10^6^ (see Figure 5 and Table 4) .

**Table 4:** Growth rates for a 10-fold dilution series of *V. parahaemolyticus* in the absence and presence of *Vp* cell-free culture fluids.

Dilution	Cell-free culture fluid Growth rate (Doublings/H)	
	-	+
1∶10	4.2±0.40	3.9±0.40
1∶10^2^	5.9±0.20	5.9±0.40
1∶10^3^	6.5±0.20	6.3±0.10
1∶10^4^	6.7±0.10	6.5±0.10
1∶10^5^	6.9±0.10	6.6±0.10
1∶10^6^	7.1±0.05	5.9±0.10

**Figure 6:**
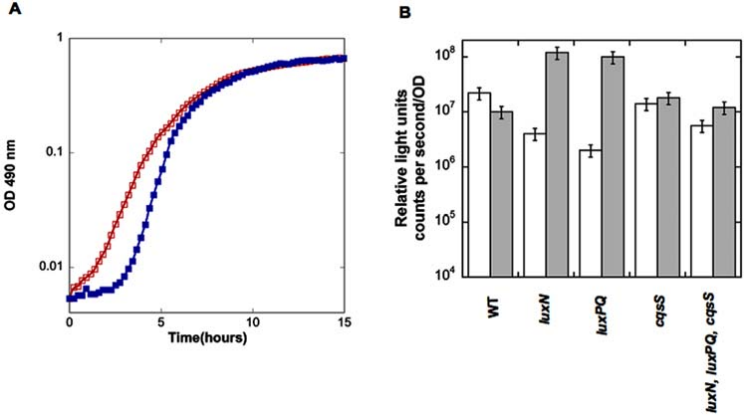
(A) Cell-free culture fluid from *Vibrio parahaemolyticus* influenced the growth rates of *Vibrio harveyi.* In these experiments, *Vh* BB120 served as the wild-type reference strain. BB120, WT in MB (○); BB120, WT in 10% *Vp* UM4552 cell-free culture fluids (•) (B) *Vh* bioluminescence phenotypes in the absence (white bars) and presence (gray bars) of *Vp* cell-free culture fluids. Strain designations are: BB120 (WT); BB170 (*luxN*); BB886 (*luxPQ*); JMH598 (*cqsS*) and JMH628 (*luxN*, *luxPQ*, *cqsS*). The accompanying table shows growth rates for each *Vh* strain in the absence and presence of 10% *Vp* (UM4552) cell-free culture fluids.

*Vp* and *Vh* are related and may occupy related [28], though probably not identical [29], ecological niches. The influence of UM4552 cell-free culture fluids on *Vh* was assayed. 10% Cell free culture-fluid from a fresh saturated culture of UM4552 was added to cultures inoculated with wild type *Vh* and quorum sensor mutants. Wild type *Vibrio harveyi* responded differently to *Vp* cell-free culture fluids than did the quorum mutants. The addition of the cell-free culture fluids led to a prolonged lag phase followed by a significantly faster growth rate of 1.5/H in the wild type *Vh* compared to a growth rate of 1/H for the wild type *Vh* in MB alone (Figure 6A). Although growth rate of the mutants was less affected than wild type, the peak luminescence for two of the mutants was increased in the presence of *Vp* cell-free culture fluids whereas wild type luminescence was not increased (Table 5; Figure 6B). Specifically, the addition of the *Vp* UM4552 cell-free culture fluids increased light production ca 50 fold in *Vh* strains possessing LuxPQ and CqsS (BB170) or LuxN and CqsS (BB886). However, only modest increases in light production occurred following addition of UM4552 fluids to mutants lacking the CAI-1 receptor CqsS (JMH598 and JMH628) (Figure 6B).

**Table 5:** Growth rates of *V.harveyi* sensor mutants in the absence and presence of 10% *V. parahaemolyticus* (UM4552) cell-free culture fluids.

Strain Name	*Vh* strain	Autoinducers Detected	Cell-free culture fluid Growth rate (Doublings/H)	
			−	+
BB120	WT	HAI-1, AI-2, CAI-1	1.0±0.03	1.5±0.02
BB170	*luxN*	AI-2, CAI-1	1.5±0.06	1.3±0.02
BB886	*luxPQ*	HAI-1, CAI-1	1.4±0.09	1.1±0.02
JMH598	*cqsS*	HAI-1, AI-2	0.9±0.09	1.2±0.02
JMH628	*luxN*, *luxPQ*, *cqsS*	none	0.5±0.04	0.7±0.04

## Discussion

Bacterial growth rates are influenced by multiple factors including nutrients, temperature, osmotic strength, pH, and oxygen concentration. Bacteria grown under optimal conditions reach maximal growth rates that vary greatly among different species and somewhat among different strains of the same species [30]. The mechanism(s) responsible for the control of growth rates and the setting of maximum rates are unknown. There are many consequences of altering growth rate and it is difficult to unambiguously disentangle causation, correlation, and effect [31].

There are indications in the literature that quorum sensing influences bacterial growth rates. A mutation in a single quorum gene, *luxS*, affects growth rates in *E. coli O157:H7* and also in *Lactobacillus reuteri* [1], [2]. However, *luxS* also has cell-autonomous roles in carbon and nitrogen metabolism [32] that could contribute to growth rate phenotypes, although this hypothesis remains to be tested. Autoinducers play a role in slowing down growth rate as a bacterial culture enters stationary phase [3].

Factors other than quorum sensing influence the relationship of luminescence and growth in *Vibrio harveyi*. We noted a familiar luminescence-growth relationship in wild-type *Vh*, i.e. an immediate decline of luminescence in suspensions upon dilution from bright overnight cultures, followed by the induction of luminescence as a consequence of the density and time-dependent accumulation of autoinducers. Media osmolarity and composition affect luminescence induction in *V. fischeri* [33]. Diluting MB broth 4-fold with artificial seawater decreased growth rates of wild type *Vibrio harveyi*. However, the overall luminescence-growth relationship was maintained (Figure S1). Other non-quorum variables that could have influenced the exponential growth rate, included the presence of antibiotics and transposons in the mutants (for details on strain construction, see [9], [22]–[24], [34]). This appears not to have been the case, because growth rates for two wild type strains differing only by insertion of the transposon, Tn5, and presence of kanamycin were identical. A kanamycin-dependent increase in lag phase was observed but exponential growth rates were not affected (Figure 2).

The interpretation of growth rate studies involving quorum sensing in luminescent bacteria is complicated by the fact that energy channeled into light emission might compete with the energy used for growth, i.e. the energy sink hypothesis. In addition, bioluminescence consumes both reducing power and oxygen [10], [12], [13], apparently competing for substrates with aerobic respiration [19]. Quorum sensing and bioluminescence mutants allowed testing of the sum of these effects in *Vibrio harveyi*. Bioluminescence in *Vh* contributes to protection against oxidative stress [19]. Using isogenic strains and confirmation by genetic complementation, it was found that the absence of an active luciferase pathway increased *Vibrio harveyi*'s growth rate by ca. 30%. The finding of a growth rate increase for the luciferase mutant is in keeping with other studies that a *luxA* mutant outcompetes against its wild type parent [35]. However, it appears that luminescence is only one part of the growth rate effect of quorum sensing observed in *Vibrio harveyi*.

Quorum sensing allows bacteria to distinguish between conditions of low and high cell density. In this study quorum sensing mutants mimicking both high and low cell density states were examined with respect to quorum sensing-growth rate relationships. The energy sink hypothesis predicts that all growth rate changes observed would mainly be due to differences in bioluminescence. Two of the mutants that were brightly luminescent (*luxO* and the triple sensor mutant, *luxN*, *luxPQ*, *cqsS*), grew at half the rate of wild type *Vibrio harveyi*. These mutants seem to support the energy sink hypothesis as well as our alternative hypothesis that mutants mimicking high cell density states might be slow growers. However, the remaining dim mutants exhibited growth rate changes in a manner that did not correlate with their respective peak bioluminescence values or cell density states (Figure 4). Part of the reason may be attributed to the nature of the phenotypes. *Vh* mutants in the CAI-1-CqsS quorum sensing system did not grow significantly differently than the wild type. Interestingly, in *Vh*, the quorum sensing input from the CAI-1-CqsS system is significantly weaker than that from the other two systems [22], which could account for our results. Previous studies had shown the strength of autoinducer signaling to be HAI-1>AI-2>CAI-1 [9], [22]–[24], [34]. Based on these studies, it was assumed that mutants mimicking low cell density states e.g. *luxM* would potentially grow faster than mutants mimicking higher cell density states e.g. *luxPQ* (no phosphorylation of *luxO* by *luxQ* and *luxN*). Surprisingly, the *luxM* (no HAI-1) and *luxN* mutants-two strains with opposite phenotypes-were our fastest-growers. In the case of the *luxM* mutation (no HAI-1), the growth phenotype could not be complemented, indicating the possibility of second-site mutations and/or complexities inherent to the plasmid-borne complementation system. The growth rate increments observed for the *luxN*, *luxPQ* and *luxS* mutations were confirmed by genetic complementation, indicating a role for these genes in affecting *Vh* growth rate by an unknown mechanism. This study adds to previous findings that a *luxS* null mutation increases the growth rate of *E. coli O157:H7* [1] and expands the findings to more quorum genes.

Genes controlled by quorum sensing have not been extensively investigated in *Vp*, although it is known that quorum sensing controls expression of a type III secretory system and the opacity-translucent transition in *Vp* [22], [36], [37]. PCR analysis suggests that the rapidly growing *Vp* isolate, UM4552, possesses the entire complement of *Vh* quorum sensing genes (Data not shown. See Table S1 for PCR primers used).

UM4552 cell-free culture fluids (from 1×MB) prolonged the lag phase and increased the growth rate of wild type *Vh* (Figure 6A). Results with cell-free culture fluids must be interpreted with caution in the sense that they might not be due to quorum factors *per se*. Growth rate enhancement by the cell-free culture fluids addition could be due to the presence of an inhibitory factor or providing a source of otherwise limiting nutrients or cofactors such as secreted siderophores [38]. Simple dilution seems unlikely since 10% volume of culture cell-free culture fluids was added. There were slight differences in growth rate upon addition of cell-free culture fluids to *Vh luxN*, *luxPQ* and *cqsS* mutants (Table 5). However, the fact that *Vp* cell-free culture fluids increased the luminescence of reporter strains responsive to CAI-1 (Figure 6B) is important for two reasons: First, the absence of a major growth rate change in strains in which luminescence has been dramatically increased provides further evidence that luminescence alone is not the primary influence of quorum sensing on growth rate in *Vibrio harveyi*. Second, the fact that mutants responsive to CAI-1 showed an increase in bioluminescence, points to a possible mechanism for understanding the effects of cell-free culture fluids from the fast-growing *Vp* strain on *Vh*. Among quorum sensing components present in all members of the Vibrio genus, there are certainly differences in alleles, and there might also be differences in gene expression levels, and relationships between genes. Possibly, *Vp* only regulates *Vh* growth rate if the *cqsS* sensor for CAI-1 is present. Alternatively, it does not make HAI-1 or AI-2, or it makes molecules with different structures that are not detected by the reporter strains, or finally *Vp* could make HAI-1 and AI-2 but at much lower quantities than does *Vh*.

The fact that *Vp* grew fastest at the highest dilution (Figure 5) is intriguing and may indicate the presence of a factor in the cell-free culture fluids that decreases growth rates at higher cell densities. The fast-growing *Vibrio parahaemolyticus* strain in this study may employ a high rate of rRNA synthesis adopted by other Vibrios to maintain a “burst” of fast growth under optimal nutrient conditions [18]. A high rate of translation or simply a lack of luciferase could also contribute to *Vp* fast growth. Why then would *Vp* need to produce signaling molecules? Autoinducers may participate in growth phase transitions to stationary phase through sensing of cell density, as has been suggested from studies of *E. coli* batch cultures [39]. Based on the fact that *Vp* cell-free culture fluids influence both *Vh* growth and luminescence, *Vp* autoinducers/diffusible factors may influence *Vh* phenotypes in the environment where they can be found in mixed-species consortia. Precedent for one strain affecting the growth of another strain via diffusible factors, comes from studies showing that a *Micrococcus luteus* proteinaceous factor, Rpf, can resuscitate dormant *Mycobacterium tuberculosis* [40].

*Vibrio harveyi* strains with wild type quorum sensing systems grew more slowly in unsupplemented marine broth. The more rapid growth of quorum mutants under laboratory conditions may have contributed to the ready isolation of quorum sensing mutants. The conditions under which positive selection for quorum sensing occurs and whether this selection occurs at the level of the individual cell [41] [42] or the population remains unclear. It has been suggested that quorum sensing provides cues between species [43]. In that context it is intriguing that *Vp* cell-free culture fluids increased the growth rate of wild type *Vh*.

There are indications that the quorum sensing pathway is linked to density-independent small molecule-signaling pathways [44]. The growth-related influences of quorum factors and other molecules observed in laboratory studies may be modified and magnified to fit many particular circumstances in nature[45], where “bacterial social networking” is hypothesized as crucial to success in different ecological contexts.

A number of “chip” studies have been carried out with the hope of elucidating the global transcriptional effects of quorum sensing [1], [46], [47]. We are not going to review them here except to note that: 1) A large number ca 6–10% of the total number of different transcripts in the cell are variously reported to be affected by quorum sensing and 2) The data and interpretations of different studies do not always agree with each other. Rather than clarification we offer a further complication. Since growth rate also influences the global transcriptome and quorum sensing influences growth rate, some of the transcriptional consequences attributed to quorum sensing are likely the indirect effects of altered growth rate. Conversely, although we have ruled out bioluminescence as the total explanation of the effects of quorum sensing on the growth rate of *Vh*, there remain hundreds of other transcriptional changes that could potentially mediate indirect effects on growth rate. It remains our hypothesis that through quorum sensing and other mechanisms, microbial cells and cultures are able to set their growth rates at levels less than those that are limiting by physiological capability, available nutrients or other environmental constraints. Growth at less than the maximum possible rate is not controversial for eukaryotic cells, particularly in the context of multicellular, differentiated organisms where unrestrained growth is an attribute of cancer. Legitimizing the concept of restrained growth in the realm of bacteria will be aided by further appreciation of conditions under which growing as fast as possible is disadvantageous to the cell and or population [48].

We speculate that the positive and negative effects of quorum sensing on the growth rate that are studied here are related to the difficulty of culturing many microbes found in nature [49] [50]. Others [51] as well as ourselves (Nackerdien, unpublished) have noted that plating a dilution series of bacteria from soil samples often yields nonlinear colony counts; more colonies arise at higher dilutions than predicted by the amount of dilution. A possible explanation for this strange efficiency of colony formation is the dilution of inhibitory factors. On the other hand, in some cases bacterial cell extracts [52], or environmental waters containing bacterial products[53] , allow the growth of otherwise-uncultivable bacteria. Low concentrations of antibiotics and other small molecule secreted by bacteria affect the transcriptional network of other microbes [54]. Net growth promotion or inhibition may be the product of multiple interactions and exotic bacteria may demand an appreciation of both positive and negative factors. Extending and specifying bacterial culture may become as demanding as the culture and differentiation of cell lines from eukaryotic multicellular organisms [55].

## Materials and Methods

*Bacterial strains and vectors* used in this study are listed in Table 1. The *Vp* strain, UM4552, was a gift from Dr. R. Colwell. Three single sensor *Vh* mutants, strain BB170 (*luxN*::Tn5, sensor1−, sensor2+, sensor3+); BB886 (*luxPQ*::Tn5, sensor1+, sensor2−, sensor3+) and JMH598 (*cqsS*::Cm^r^, sensor1+, sensor2+, sensor3−) were also used for *Vp* extracellular factor activity assays. Standard microscopy was done routinely to check for the possibility of changes in cell morphology or aggregates affecting OD readings.

*Cell-free culture fluids for Vp extracellular factor activity assays* were prepared by centrifugation at 5000 *g* for 10 minutes and filtration of the fluids through 0.2 µm-pore-size membrane filter units (Nalgene Labware Division, Nalge/Sybron Corp). *Vp* extracellular factor activities are reported as light output from *Vh* sensor mutants alone versus *Vh* strains supplemented with 10% cell-free culture fluids (in filtered Difco Marine Broth).

### Growth rates and bioluminescence were determined as follows

Growth rates for flask cultures of wild type *Vp* and *Vh* (strain BB120) were first determined by standard spectrophotometry and analyzed by the slopes of best-fit lines to the mid-exponential phase region of the growth curves. These growth rates were comparable to growth rates from automated assays (data not shown). To obtain rapid and quantitative analyses of Vibrio strain sets, we inoculated 96 well-microtiter plates containing MB (total volume = 150 µl) with pre-cultures of *Vh* and *Vp* (age of cultures ≤ 10 h) that had been incubated at 30°C and 37°C, respectively. In the case of *Vh* strains OD and bioluminescence measurements were taken every 10 minutes. For the faster growing *Vp* stain OD was measured every 2.5 minutes. Both cell growth and bioluminescence were measured in triplicate with background taken into account. Light output is reported as Relative Light Units (RLU) i.e. counts per second divided by OD. Standard deviations are given in the Results section. Output data from a Victor Wallac III Multilabel Plate Reader (Perkin Elmer, California) was imported into Kaleidagraph (Synergy, PA).

### DNA manipulations were performed according to standard protocols [56]

PCR conditions: 94°C for 2 min, 94°C 30 sec, 60°C 40 sec, 72°C 30 sec, repeat step 2-step 4 40X. Table S1 provides a summary of primer sets used to test for quorum genes in *Vp*.

## Supporting Information

Table S1 Primer sets used to document quorum sensing genes Vp 4552
(0.05 MB DOC)

Figure S1 Bioluminescence/Growth relationship of Vibrio harveyi changes with growth medium. The bioluminescence-growth relationships of wild type Vh upon diluting Difco Marine Broth (MB) with artificial seawater (ASW). (A) Strain BB120, MB:ASW = 1:2 (Growth Rate Open Squares, Filled Squares Bioluminescence. Relative Light Units (RLU) expressed as counts per second. (B) ) Strain BB120, MB:ASW = 1:4 (GR Open Triangles, Filled Triangles Bioluminescence; (C) Strain BB866 (WT::Tn5), MB:ASW = 1:2 (Open Squares Growth Rate, Filled Squares Bioluminescence; (D) Strain BB866, MB:ASW = 1:4 (Growth Rate Open Triangle, Filled Triangle Bioluminescence.
(2.24 MB TIF)

## References

[1] Sperandio V, Torres AG, Giron JA, Kaper JB (2001). Quorum sensing is a global regulatory mechanism in enterohemorrhagic Escherichia coli O157:H7.. J Bacteriol.

[2] Tannock GW, Ghazally S, Walter J, Loach D, Brooks H (2005). Ecological behavior of Lactobacillus reuteri 100-23 is affected by mutation of the luxS gene.. Appl Environ Microbiol.

[3] Lazazzera BA (2000). Quorum sensing and starvation: signals for entry into stationary phase.. Curr Opin Microbiol.

[4] Shapiro J, Dworkin M (1997). Bacteria as Multicellular Organisms..

[5] Tomasz A (1965). Control of the competent state in Pneumococcus by a hormone-like cell product: an example for a new type of regulatory mechanism in bacteria.. Nature.

[6] Surette MG, Miller MB, Bassler BL (1999). Quorum sensing in Escherichia coli, Salmonella typhimurium, and *Vibrio harveyi*: a new family of genes responsible for autoinducer production.. Proc Natl Acad Sci U S A.

[7] Swift S, Throup JP, Williams P, Salmond GP, Stewart GS (1996). Quorum sensing: a population-density component in the determination of bacterial phenotype.. Trends Biochem Sci.

[8] Mok KC, Wingreen NS, Bassler BL (2003). *Vibrio harveyi* quorum sensing: a coincidence detector for two autoinducers controls gene expression.. Embo J.

[9] Bassler BL, Greenberg EP, Stevens AM (1997). Cross-species induction of luminescence in the quorum-sensing bacterium *Vibrio harveyi*.. J Bacteriol.

[10] Nealson KH, Platt T, Hastings JW (1970). Cellular control of the synthesis and activity of the bacterial luminescent system.. J Bacteriol.

[11] Nealson KH, Hastings JW (1979). Bacterial bioluminescence: its control and ecological significance.. Microbiol Rev.

[12] Hastings JW, Nealson KH (1977). Bacterial bioluminescence.. Annu Rev Microbiol.

[13] Hastings JW, Greenberg EP (1999). Quorum sensing: the explanation of a curious phenomenon reveals a common characteristic of bacteria.. J Bacteriol.

[14] Ingraham J, Maaloe O, Neidhardt F (1983). Growth of the Bacterial Cell..

[15] Neidhardt FC (1999). Bacterial growth: constant obsession with dN/dt.. J Bacteriol.

[16] Brauer MJ, Saldanha AJ, Dolinski K, Botstein D (2005). Homeostatic adjustment and metabolic remodeling in glucose-limited yeast cultures.. Mol Biol Cell.

[17] Karlin S, Mrazek J, Campbell A, Kaiser D (2001). Characterizations of highly expressed genes of four fast-growing bacteria.. J Bacteriol.

[18] Aiyar S, Gaal T, Gourse RL (2002). rRNA promoter activity in the fast-growing bacterium *Vibrio natriegens*.. J Bacteriol.

[19] Stabb EV (2005). Shedding light on the bioluminescence paradox.. ASM News.

[20] Lupp C, Ruby EG (2004). *Vibrio fischeri* LuxS and AinS: comparative study of two signal synthases.. J Bacteriol.

[21] Higgins DA, Pomianek ME, Kraml CM, Taylor RK, Semmelhack MF (2007). The major Vibrio cholerae autoinducer and its role in virulence factor production.. Nature.

[22] Henke JM, Bassler BL (2004). Three parallel quorum systems regulated gene expression in *Vibrio harveyi*.. J Bacteriol.

[23] Freeman JA, Bassler BL (1999). A genetic analysis of the function of LuxO, a two-component response regulator involved in quorum sensing in *Vibrio harveyi*.. Mol Microbiol.

[24] Bassler BL, Wright M, Silverman MR (1994). Multiple signalling systems controlling expression of luminescence in *Vibrio harveyi*: sequence and function of genes encoding a second sensory pathway.. Mol Microbiol.

[25] Blot M, Hauer B, Monnet G (1994). The Tn5 bleomycin resistance gene confers improved survival and growth advantage on Escherichia coli.. Mol Gen Genet.

[26] Chatterjee J, Miyamoto CM, Zouzoulas A, Lang BF, Skouris N (2002). MetR and CRO bind to the *Vibrio harveyi* lux promoters and regulate luminescence.. MolMicrobiol.

[27] Stabb EV, Butler MS, Adin DM (2004). Correlation between osmolarity and luminescence of symbiotic *Vibrio fischeri* strain ES114.. JBacteriol.

[28] Henke JM, Bassler BL (2004). Quorum sensing regulates type III secretion in *Vibrio harveyi* and *Vibrio parahaemolyticus*.. J Bacteriol.

[29] Simidu U, Tsukamoto K (1985). Habitat Segregation and Biochemical Activities of Marine Members of the Family Vibrionaceae.. Appl Environ Microbiol.

[30] Beste DJ, Laing E, Bonde B, Avignone-Rossa C, Bushell ME (2007). Transcriptomic analysis identifies growth rate modulation as a component of the adaptation of mycobacteria to survival inside the macrophage.. J Bacteriol.

[31] Bettenbrock K, Sauter T, Jahreis K, Kremling A, Lengeler JW (2007). Correlation between growth rates, EIIACrr phosphorylation, and intracellular cyclic AMP levels in Escherichia coli K-12.. J Bacteriol.

[32] Kendall MM, Rasko DA, Sperandio V (2007). Global effects of the cell-to-cell signaling molecules autoinducer-2, autoinducer-3, and epinephrine in a luxS mutant of enterohemorrhagic Escherichia coli.. Infect Immun.

[33] Stabb EV, Butler MS, Adin DM (2004). Correlation between osmolarity and luminescence of symbiotic *Vibrio fischeri* strain ES114.. J Bacteriol.

[34] Bassler BL, Wright M, Showalter RE, Silverman MR (1993). Intercellular signalling in *Vibrio harveyi*: sequence and function of genes regulating expression of luminescence.. Mol Microbiol.

[35] Czyz A, Plata K, Wegrzyn G (2003). Stimulation of DNA repair as an evolutionary drive for bacterial luminescence.. Luminescence.

[36] McCarter LL (1998). OpaR, a homolog of *Vibrio harveyi* LuxR, controls opacity of *Vibrio parahaemolyticus*.. J Bacteriol.

[37] Jaques S, McCarter LL (2006). Three new regulators of swarming in *Vibrio parahaemolyticus*.. J Bacteriol.

[38] Lilley BN, Bassler BL (2000). Regulation of quorum sensing in *Vibrio harveyi* by LuxO and Sigma-54.. MolMicrobiol.

[39] Carbonell X, Corchero JL, Cubarsi R, Vila P, Villaverde A (2002). Control of Escherichia coli growth rate through cell density.. Microbiol Res.

[40] Kaprelyants AS, Kell DB (1993). Dormancy in Stationary-Phase Cultures of Micrococcus luteus: Flow Cytometric Analysis of Starvation and Resuscitation.. Appl Environ Microbiol.

[41] Redfield RJ (2002). Is quorum sensing a side effect of diffusion sensing?. Trends Microbiol.

[42] Turovskiy Y, Kashtanov D, Paskhover B, Chikindas ML (2007). Quorum sensing: fact, fiction, and everything in between.. Adv Appl Microbiol.

[43] Diggle SP, Gardner A, West SA, Griffin AS (2007). Evolutionary theory of bacterial quorum sensing: when is a signal not a signal?. Philos Trans R Soc Lond B Biol Sci.

[44] Camilli A, Bassler BL (2006). Bacterial small-molecule signaling pathways.. Science.

[45] Jacob F (1977). Evolution and Tinkering.. Science.

[46] Zhu J, Miller MB, Vance RE, Dziejman M, Bassler BL (2002). Quorum-sensing regulators control virulence gene expression in Vibrio cholerae.. Proc Natl Acad Sci U S A.

[47] Vasil ML (2003). DNA microarrays in analysis of quorum sensing: strengths and limitations.. J Bacteriol.

[48] Lederberg J (1957). Mechanism of action of penicillin.. J Bacteriol.

[49] Colwell R, Grimes D (2000). Nonculturable Microorganisms in the Environment..

[50] Rappe M, Giovannoni S (2003). The uncultured microbial majority.. Ann Rev Microbiol.

[51] Davis K, Joseph S, Janssen P (2005). Effects of growth medium, inoculum size, and incubation time on culturability and isolation of soil bacteria.. Appl Environ Microbiol.

[52] Lederberg J, St. Clair J (1958). Protoplasts and L-type growth of Escherichia coli.. J Bact.

[53] Kaeberlein T, Lewis K, Epstein SS (2002). Isolating “uncultivable” microorganisms in pure culture in a simulated natural environment.. Science.

[54] Yim G, Wang HH, Davies J (2007). Antibiotics as signalling molecules.. Philos Trans R Soc Lond B Biol Sci.

[55] Freshney R (2005). Culture of Animal Cells: A Manual of Basic Technique:.

[56] Sambrook J, Fritsch EF, Maniatis T (1989). Molecular cloning a laboratory manuel..

[57] Ditta G, Stanfield S, Corbin D, Helinski DR (1980). Broad host-range cloning system for gram negative bacteria:construction of a gene bank for Rhizobium melilotti.. ProcNatlAcadSciUSA.

[58] Makino K, Oshima K, Kurokawa K, Yokoyama K, Uda T (2003). Genome sequence of *Vibrio parahaemolyticus*: a pathogenic mechanism distinct from that of *V cholerae*.. Lancet.

