# Supplementary material for: Quorum Sensing Influences Vibrio harveyi Growth Rates in a Manner Not Fully Accounted For by the Marker Effect of Bioluminescence
Source: PLoS One. 2008 Feb 27;3(2):e1671. doi: 10.1371/journal.pone.0001671 (PMC2249925; doi:10.1371/journal.pone.0001671)
Supplement: Table S1 — Primer sets used to document quorum sensing genes Vp 4552 (0.05 MB DOC) [file pone.0001671.s001.doc]

Table S1 shows primer sets used for *Vp*. Accession numbers are given in brackets and based on the sequenced strain RIMD221063 [58] .

| Primer | Sequence (5 ‘→ 3 ’) |
| --- | --- |
| *luxM* [VP1967] | TGGCAAGTGACCTCGTTACAGACT  ACCCAACCTTGTAGCCTGACTGAA |
| *luxS* [VP2537] | GGGCCACCCAATGGAAATTGTTGA  TGTTAGGCGCAGTGAAGCGTAAGT |
| *cqsA* [VPA0711] | AAGCGCATCGAGACGAATTTCTG  AAGGTTTGGAGCGCAGATGGTTTG |
| *opaR* [VP2516] | TCGCGTTAGAAGTATTTGCACGCC  TGGTTAGTGCGGTTGGTAGACACA |
| *luxN* [VP1968] | GCACCACTGTGCTCATTGTCGAT  CGCTGGCATCAAACCCATTCATCA |
| *luxP* [VPA1221] | TGTTCTACCGACGTTGCGTTAGGT  TCCCATTTGATCGCCTCTGCCATA |
| *luxQ* [VPA1220] | AACGTCGACAATGTTGTGAAGTGA  ACACTCTGGTTGTCTTGCACGGTA |
| *cqsS* [VPA0710] | ACGTAACCTTGCTCCGTGAAGTGA  TCGAATGAGATCGCGCAACCTGTCT |
| *luxO* [VP2099] | GGTCAACAACGCAATTCGCAAAGC  TTCTGCGCATACCTCTTTACCCGT |
| *luxU* [VP2098] | TCGGCAGTGATAACGTGCCTGTTT  GCTAACTCACACACAAACTGTCGGCT |
