# Supplementary figures and images for: Quorum Sensing Influences Vibrio harveyi Growth Rates in a Manner Not Fully Accounted For by the Marker Effect of Bioluminescence
Source: PLoS One. 2008 Feb 27;3(2):e1671. doi: 10.1371/journal.pone.0001671 (PMC2249925; doi:10.1371/journal.pone.0001671)

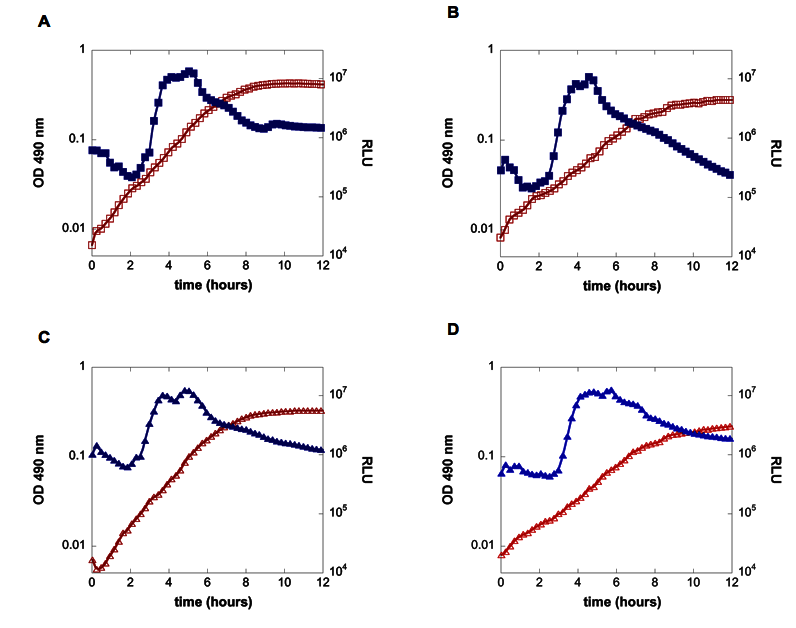

Supplement: Figure S1 — Bioluminescence/Growth relationship of Vibrio harveyi changes with growth medium. The bioluminescence-growth relationships of wild type Vh upon diluting Difco Marine Broth (MB) with artificial seawater (ASW). (A) Strain BB120, MB:ASW = 1:2 (Growth Rate Open Squares, Filled Squares Bioluminescence. Relative Light Units (RLU) expressed as counts per second. (B) ) Strain BB120, MB:ASW = 1:4 (GR Open Triangles, Filled Triangles Bioluminescence; (C) Strain BB866 (WT::Tn5), MB:ASW = 1:2 (Open Squares Growth Rate, Filled Squares Bioluminescence; (D) Strain BB866, MB:ASW = 1:4 (Growth Rate Open Triangle, Filled Triangle Bioluminescence. (2.24 MB TIF) [file pone.0001671.s002.tif]
